# Assessment of the impact of inpatient infectious events in pediatric patients with newly diagnosed acute leukemia at Dr. Robert Reid Cabral Children’s Hospital, Dominican Republic

**DOI:** 10.1371/journal.pone.0243795

**Published:** 2020-12-15

**Authors:** Julianne E. Burns, Dominga Reyes Pérez, Yimei Li, Wendy Gómez García, F. Jay Garcia, Johanna Penélope Gil Jiménez, Jacqueline Sánchez, María Castillo Bueno, Stephen P. Hunger, Lisa Reaves, Johanny Contreras González, Susan E. Coffin, Adriana Deverlis, Andrew P. Steenhoff, Brian T. Fisher

**Affiliations:** 1 Division of Infectious Diseases, Department of Pediatrics, Children’s Hospital of Philadelphia, Philadelphia, Pennsylvania, United States of America; 2 Division of General Pediatrics, Department of Pediatrics, Children’s Hospital of Philadelphia, Philadelphia, Pennsylvania, United States of America; 3 Department of Pediatrics, Perelman School of Medicine, University of Pennsylvania, Philadelphia, Pennsylvania, United States of America; 4 Department of Oncology, Hospital Infantil Dr. Robert Reid Cabral, Santo Domingo, National District, Dominican Republic; 5 Department of Biostatistics, Epidemiology, and Informatics, Perelman School of Medicine, University of Pennsylvania, Philadelphia, Pennsylvania, United States of America; 6 Division of Oncology and the Center for Childhood Cancer Research, Department of Pediatrics, Children’s Hospital of Philadelphia, Philadelphia, Pennsylvania, United States of America; 7 Microbiology Laboratory, Department of Infectious Diseases, Hospital Infantil Dr. Robert Reid Cabral, Santo Domingo, National District, Dominican Republic; 8 Center for Cancer and Blood Disorders, Children’s Hospital Colorado, Colorado Springs, Colorado, United States of America; 9 Global Health Center, Children’s Hospital of Philadelphia, Philadelphia, Pennsylvania, United States of America; Universita degli Studi di Parma, ITALY

## Abstract

Survival rates for pediatric acute leukemia vary dramatically worldwide. Infections are a leading cause of morbidity and mortality, and the impact is amplified in low and middle-income countries. Defining the epidemiology of infection in a specific health care setting is paramount to developing effective interventions. This study aimed to define the epidemiology of and outcomes from infection in children with acute leukemia treated in a large public pediatric hospital in the Dominican Republic. A retrospective cohort was assembled of children newly diagnosed with acute leukemia between July 1, 2015 to June 30, 2017 at Hospital Infantil Dr. Robert Reid Cabral in Santo Domingo. Patients were identified from the Pediatric Oncology Network Database (POND^TM^) and hospital admissions from the Oncology admissions logbook. Medical records and microbiology results were reviewed to identify all inpatient invasive infections. Distance from a child’s home to the hospital was determined using ArcGIS by Esri. Infection rates were described in discrete time periods after diagnosis and risk factors for invasive infection were explored using negative binomial regression. Overall, invasive infections were common and a prominent source of death in this cohort. Rates were highest in the first 60 days after diagnosis. Gastroenteritis/colitis, cellulitis, and pneumonia were most frequent, with bacteremia common early on. Multidrug resistant bacteria were prevalent among a small number of positive cultures. In a multivariate negative binomial regression model, age ≥ 10 years and distance from the hospital > 100 km were each protective against invasive infection in the first 180 days after diagnosis, findings that were unexpected and warrant further investigation. Over one-third of patient deaths were related to infection. Interventions aimed at reducing infection should target the first 60 days after diagnosis, improved supportive care inside and outside the hospital, and increased antimicrobial stewardship and infection prevention and control measures.

## Introduction

Infection is a leading cause of treatment-related morbidity and mortality among pediatric patients diagnosed with cancer throughout the world. Infection-related mortality is further amplified in low- and middle-income countries (LMIC), with rates up to 10 times higher than in high-income countries (HIC) [1, 2]. This inequity in infection outcomes is a primary reason why overall cancer survival in LMIC lags far behind those in HIC [2]. Thus, identification of effective interventions aimed at reducing infection-related morbidity and mortality will improve a child’s opportunity to survive their cancer. In a model simulating childhood cancer survival worldwide, improving the quality of care (supportive services and facility-level activities, including infection control) yielded the largest potential survival gains among individual policy interventions [3].

Unfortunately, the source of infection-related morbidity and mortality varies between geographic locations and even between different institutions in a similar geographic location. This is likely related to variation in epidemiology of infectious pathogens and variability in access to resources to prevent, recognize, and treat infection. Therefore, there is not a single approach that can be implemented across institutions in LMIC to effect improvements. Instead it is paramount to first define the epidemiology of and risk factors for infection in a specific setting. This knowledge can then be used to inform development and implementation of effective interventions.

This study aims to define the epidemiology of, risk factors for, and outcomes from infections among hospitalized children with newly diagnosed acute leukemia at Hospital Infantil Dr. Robert Reid Cabral (HIRRC), the largest children’s hospital in the Dominican Republic and the primary provider of oncologic care for a large population of children in the southern half of the country. We focused on infections diagnosed in the inpatient setting, given a greater likelihood of severity and impact on clinical course.

## Methods

### Study approval

This study was approved by the institutional review boards of the Children’s Hospital of Philadelphia (IRB 17–014329) and Hospital Infantil Dr. Robert Reid Cabral (IRB 3508, FWA 4747), as well as the Dominican Republic’s national institutional review board—Consejo Nacional de Bioética en Salud (CONABIOS 003–2018). A waiver of consent/parental permission and a waiver of assent was approved given the retrospective nature of the study and that adequate provisions were in place to protect the confidentiality of the data.

### Study design and cohort assembly

A retrospective cohort was assembled consisting of all children less than 17 years of age newly diagnosed with acute lymphoblastic (ALL) or acute myeloid leukemia (AML) between July 1, 2015 to June 30, 2017 at HIRRC. HIRRC is a pediatric hospital with approximately 370 inpatient beds. Patients were identified by date of diagnosis through the Pediatric Oncology Network Database (POND^TM^), a secure, web-based pediatric hematology/oncology database established to capture clinical data on oncologic patients in LMIC. POND is a component of the global medicine program at St. Jude Children’s Research Hospital [4]. This database contains information on demographics, cancer diagnosis and treatment, including date of diagnosis, type of cancer diagnosed, and treatment protocol. The database served as a source of cohort assembly, but multiple additional data sources were leveraged for complete data capture as described below.

### Data capture

The Oncology unit admissions logbook was reviewed to identify all admissions of patients in the cohort within the two-year study period. After identification of all admissions, the paper medical records were retrieved from storage for comprehensive data capture. Any admission with a missing record was documented. Two study investigators (J.B. and F.J.G.), both fluent in Spanish, performed the chart abstraction. When handwriting in the paper charts was unclear, other members of the study team were consulted until a consensus was obtained. To supplement data contained in the inpatient paper charts, results from the Oncology unit culture logbooks and the Microbiology Laboratory logbooks for the corresponding study period were also reviewed. No information that occurred on hospital days outside the two-year study period (before July 1, 2015 or after June 30, 2017) was collected from the inpatient charts, logbooks or POND. Abstracted data were directly entered into a REDCap^TM^ (Research Electronic Data Capture) database hosted at the Children’s Hospital of Philadelphia (CHOP), under an agreement with the software’s development consortium. REDCap is a secure, web-based application designed to support data capture for research studies [5]. The REDCap database was accessed through a secure web-based password protected portal. Once data abstraction was complete, data were downloaded from REDCap and imported into STATA 15.1 (Stata Corp LLC, College Station, Texas) for analysis.

### Outcomes

The primary outcome was number of invasive infections in the first 180 days after diagnosis and secondary outcomes included number of non-invasive infections in the first 180 days, time to the first invasive infection, and mortality. Invasive infection was defined as a systemic infection or infection of a specific organ system(s). This included bacteremia/candidemia or other systemic infection (e.g. dengue), pyelonephritis, meningitis, pneumonia, cellulitis, peritonitis, gastroenteritis, colitis, or typhlitis. Clinical diagnoses were supported by reports from radiographic studies and microbiologic diagnostic testing, including bacterial, viral, and parasite tests, when available. Attainment of repeat cultures after initial positivity was rare; however, when a patient did have positive repeat cultures within seven days it was counted as a single bacteremia event. Susceptibility results for positive bacterial cultures were performed by disk diffusion (Kirby-Bauer). Direct diagnostic testing for detection of carbapenemases and extended spectrum beta-lactamases (ESBL) was not available. However, an ESBL phenotype was inferred by a Modified Double-Disk test. On a plate growing the test isolate, an amoxicillin-clavulanic acid disk is placed between cefotaxime and ceftazidime disks (20 mm from center to center on each side), and in the presence of an ESBL there is augmentation of the zone of inhibition towards the amoxicillin-clavulanic acid disk as clavulanic acid resists the ESBL (described as the “egg effect”). Non-invasive infections were categorized broadly as infections that were limited to involvement of mucosal barriers, including upper respiratory tract infections, otitis media, infectious stomatitis, cutaneous *Herpes simplex* and *zoster*, candidal dermatitis, and ocular infections (e.g. conjunctivitis). Bronchospasm and wheezing that were not described as having a clear infectious trigger were not categorized as infections. Events of scabies and tinea capitis/corporis were not collected due to presumed lack of morbidity.

For each recorded infection, date of infection was listed as the date that the infection was first clinically diagnosed in a progress note, or the date a microbiologically positive sample was obtained, whichever came first. If the infection was diagnosed within the 10 days prior to acute leukemia diagnosis (within the same admission), the infection start date was defined as the date of acute leukemia diagnosis. This decision was founded on the assumption that the infection and acute leukemia onset were likely to be concurrent given the time required to make an acute leukemia diagnosis. Absolute neutrophil count (ANC) at the time of infection was determined by multiplying the total white blood count by the proportion of neutrophils (inclusive of segmented neutrophils and bands).

To assess inter-abstractor consistency regarding documented infections, 40 admissions were reviewed in duplicate (20 charts abstracted by J.B. were reviewed by F.J.G., and vice-versa) and correlation of captured infections were compared between the two reviewers.

With respect to the secondary outcome mortality, if a patient died during an admission, the paper medical record from that admission was sent to a hospital epidemiology office separate from routine medical record storage. Retrieval of these records was attempted; however, only three records were available owing to damage to the office related to flooding from a tropical storm. For the remaining inpatient deaths, the POND database was utilized to abstract primary and secondary causes of death and any notes related to the cause of death in that database. If an infection was described in association with the cause of death from the POND database, then that infection was captured as a primary outcome and a date of onset of seven days prior to death was assumed for that infection.

### Covariates

Captured baseline covariates included the following: acute leukemia type, sex, age and weight-for-age percentile at diagnosis, distance from home residence to hospital (HIRRC), and purchasing power index and average household size by municipality. Proportions were calculated for categorical variables, and medians and ranges for continuous variables. Age categorizations were chosen based on age stratifications of ALL risk that would be expected to alter infection risk as well. Weight obtained closest to diagnosis date was used to determine weight-for-age percentile, calculated using SAS 9.3 (SAS Institute Inc., Cary, NC), utilizing programs available from the Centers for Disease Control and Prevention (CDC). These programs are based on the 2000 CDC Growth Charts for ages 2 to < 20 years [6] and 2006 World Health Organization (WHO) Growth Charts for ages 0 to < 2 years [7]. Classification was based on recommended body mass index (BMI) for age cutoffs, with overweight and obese ≥ 85^th^ percentile, healthy weight 5^th^ to < 85^th^ percentile, and underweight < 5^th^ percentile [8]. City of residence of each patient was mapped using ArcGIS Online (2019) by Esri (Environmental Systems Research Institute, Inc., Redlands, CA). Distance from the hospital in kilometers was calculated using existing roads. Distance categories were determined based on an empiric approach to encapsulate the geography of the region, with a radius of 30 km encompassing the greater Santo Domingo area, and a radius of 100 km encapsulating the next group of outlying cities. It was estimated that transportation to the hospital from greater than 100 km away would take a significant amount of time. This advanced travel time could be a deterrent to seeking prompt care or could allow for an infection to worsen while in transit. Average number of residents per household and purchasing power index, corresponding to the disposable income of private households, was determined by municipality per 2016 data from Michael Bauer Research (Germany), available in ArcGIS Online. Purchasing power index categorizations were created based on a 20% deviation in either direction from the national average. The map of the Dominican Republic with each location of residence was finalized in ArcGIS 10.5.1, and color-coded depending on whether a patient experienced at least one invasive infection in the first 180 days after diagnosis.

### Chemotherapy regimens and supportive care practices

The ALL patients were treated with modified versions of Regimens 3 and 4 [9], which consist of induction, consolidation, interim maintenance, delayed intensification, and maintenance phases. The four-week induction phase includes prednisone, vincristine, L-asparaginase, and intrathecal methotrexate without an anthracycline. Post-induction treatment intensity was based on clinical features and early morphological response. Standard risk patients had B-ALL with age 1–9.99 years, initial white blood cell count <50,000/microliter, no central nervous system or testicular leukemia, a good response to a prednisone prophase, <25% marrow blasts at day 15 and <5% at day 29. All other patients were high-risk. Standard-risk patients received a four-week consolidation phase with weekly vincristine, 6-mercaptopurine (MP), and intrathecal methotrexate, an eight-week interim maintenance phase with monthly 5-day dexamethasone pulses with vincristine, daily oral 6-MP and weekly oral methotrexate, with intrathecal methotrexate. High-risk patients received a 6-week consolidation with intravenous cyclophosphamide (day 1 and 15), cytarabine given on days 1–4, 8–11, 15–18, 22–25, oral 6-MP days 1–28, and weekly intrathecal methotrexate, followed by an 8-week interim maintenance with 4 courses of high dose methotrexate (2 gm/m^2^) plus leucovorin rescue given every 2 weeks. All patients received an eight-week delayed intensification phase with dexamethasone, vincristine, doxorubicin, L-asparaginase, cyclophosphamide, cytarabine, 6-MP, and intrathecal methotrexate, followed by maintenance therapy with daily oral 6-MP, weekly oral methotrexate, every 4-week 5-day dexamethasone plus vincristine pulses, and intrathecal methotrexate every 12 weeks. Cranial irradiation was given to patients with central nervous system involvement at diagnosis and select other patients.

AML patients were treated with the AHOPCA AML regimen (-BFM adapted 2012) [10]. In this regimen, the induction phase consists of cytarabine, daunorubicin, etoposide, and methotrexate, with a stratified second induction phase and third induction phase if poor response. Consolidation includes Ara-C, mitoxantrone, and intrathecal methotrexate, hydrocortisone, and cytarabine, with stratified second, third, and fourth phases depending on response. There is also weekly intrathecal chemotherapy with methotrexate, hydrocortisone, and cytarabine for patients with central nervous system disease.

The local supportive care practices during the study period included trimethoprim/sulfamethoxazole for *Pneumocystis jiroveci* prophylaxis, divided into two daily doses for three consecutive days per week. In children with AML receiving chemotherapy inclusive of high doses of cytarabine, intravenous vancomycin and oral ciprofloxacin (intravenous if admitted) were administered until the ANC was greater than 500/mm^3^ and rising. Additionally, for patients with AML, during induction chemotherapy and with each course inclusive of high dose cytarabine, fluconazole and trimethoprim/sulfamethoxazole prophylaxis were given until the ANC was greater than 500/mm^3^ and rising. Patients in the cohort did not routinely have central lines placed, unless intravenous access was particularly problematic.

### Statistical analysis

Infection incidence rates were estimated as the number of infections per 100 person days. Incidence rates were described in discrete time periods (0 to 60 days, > 60 to 100 days, and > 100 to 180 days) after diagnosis. For invasive infections within the first 180 days of diagnosis, median ANC at the time of infection for each discrete time period was calculated. Kaplan-Meier survival curves were used to display time to first invasive infection and overall survival of the cohort from date of acute leukemia diagnosis. Number of days from hospital admission to infection onset was determined for all invasive infections and reported with descriptive statistics. Community-onset infection was defined as an infection occurring within the first two days of hospitalization, and hospital-acquired infection as occurring more than two days afterward.

Risk factors for number of invasive infections were explored using negative binomial regression, which accounts for differential person time at risk and potential overdispersion. The association of a risk factor with outcome was reported as an incidence rate ratio (IRR) with 95% confidence interval. Only variables that were significant (p-value < 0.05) in univariate analyses were retained in the multivariate model.

## Results

### Cohort characteristics

There were 70 patients with newly diagnosed leukemia; two patients with chronic myeloid leukemia were excluded. Of the remaining 68 patients with acute leukemia, 54 (79.4%) had ALL and 14 (20.6%) had AML. The patients were 51.5% male, with a median age at diagnosis of 7.3 years (range 1.1–16.6). There was a minority (8.8%) of children that presented with significant comorbidities. This included one each with malnutrition, trisomy 21, Acquired Immunodeficiency Syndrome, sickle cell anemia, nephrotic syndrome, and AML. For the latter patient, their current acute leukemia diagnosis was secondary ALL. Weight was available for 59 (86.8%) patients, with median weight-for-age of 45.5 percentile (range 0–99.9) [Table 1]. Location of residence was available for 67 patients (98.5%). This was listed by city, except for three patients where only province was available. In these cases, ArcGIS placed the location of residence near the center of the province. The median distance from city or province of residence to the hospital was 22.1 km (range 4.9–302.4). The median purchasing power index for the city of residence was 99.3 (range 49.3–184.4), close to the national average [Table 2]. The median average household size was 3.5 (range 3.1–4.7) persons, which was the same as the national average.

**Table 1:** Demographics.

Acute leukemia type (n = 68)	n (%)
**ALL**	**54 (79.4)**
B-cell	43 (79.6)
T-cell	8 (14.8)
Missing type	3 (1.9)
**AML**	**14 (20.6)**
M0	1 (7.1)
M1	1 (7.1)
M2	3 (21.4)
M3	4 (28.6)
M4	3 (21.4)
M5	1 (7.1)
Missing type	1 (7.1)
**Sex**	
Male	35 (51.5)
**Age at diagnosis** (years)	
Median, range	7.3, 1.1–16.6
≥ 1 to < 2 years	7 (10.3)
≥ 2 to < 10 years	32 (47.1)
≥ 10 years	29 (42.7)
**Weight-for-age percentile at diagnosis**^a^	
Median, range	45.5, 0–99.9
≥ 85^th^ (overweight & obese)	6 (8.8)
5^th^ to < 85^th^ (healthy weight)	46 (67.6)
< 5^th^ (underweight)	7 (10.3)
Missing weight	9 (13.2)

^a^For eight patients, the closest weight was > three weeks from diagnosis date (range –29 to 95 days, with three weights > 60 days).

ALL, Acute lymphoblastic leukemia; AML, Acute myeloid leukemia

**Table 2:** Location-specific demographics for home residence.

Distance to hospital (km)	n (%)
Median, range	22.1, 4.9–302.4
> 4 to 30 km	37 (54.4)
> 30 to 100 km	13 (19.1)
> 100 km	17 (25.0)
Missing	1 (1.5)
**Purchasing power index by municipality**^b^	
Median, range	99.3, 49.3–184.4
< 80	18 (26.5)
≥ 80 to ≤ 120	18 (26.5)
> 120	31 (45.6)
Missing	1 (1.5)

^b^An index of 100 indicates that the purchasing power of an area is in line with the national average, with an index above or below representing the amount of deviation from the average (email correspondence with Christiane Betzner, Division Manager Sales at Michael Bauer Research GmbH).

### Admissions and availability of medical records

**Fig 1:**
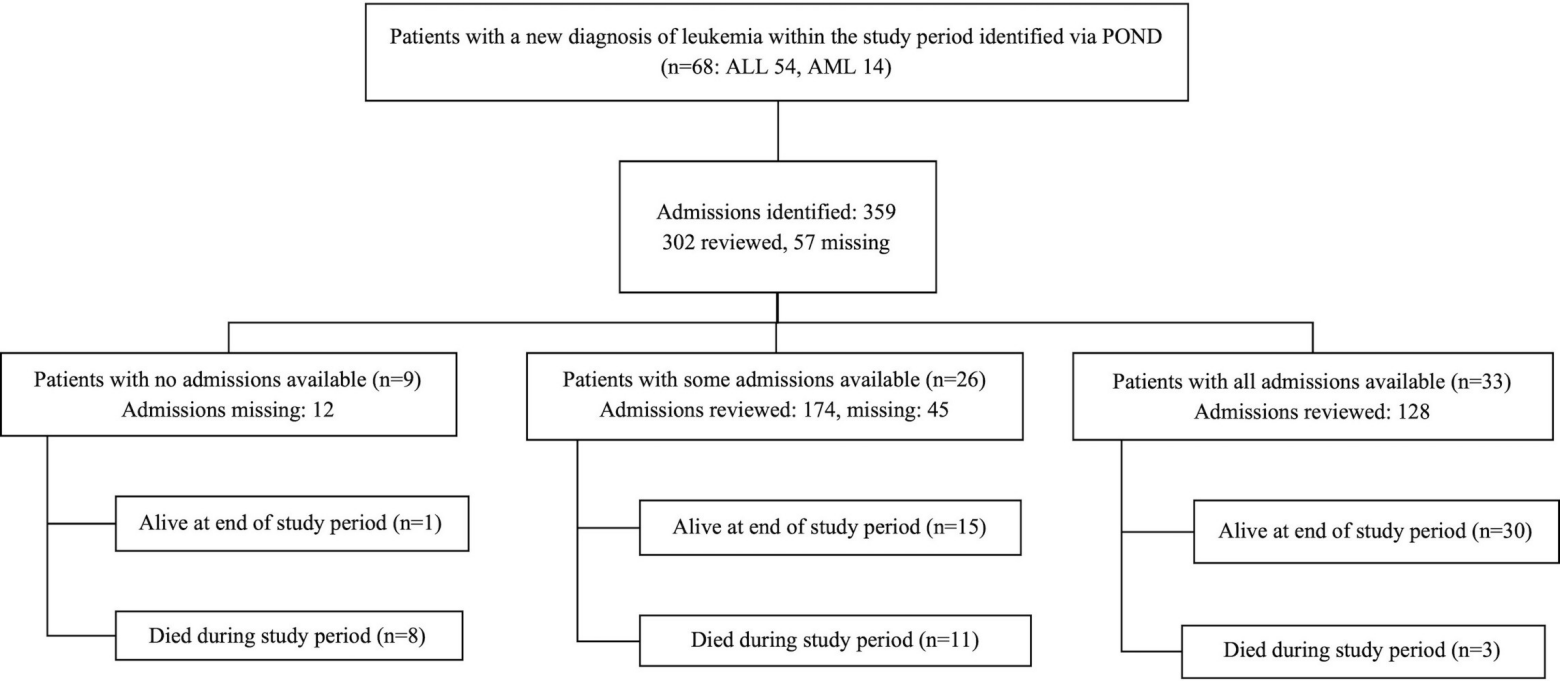
Available admission records. POND, Pediatric Oncology Network Database; ALL, Acute lymphoblastic leukemia; AML, Acute myeloid leukemia.

There were 359 total admissions for 68 patients in the cohort during the study period. Medical records were identified for 302 (84.1%) of the 359 admissions. Of the 68 patients, 33 had all admission medical records identified, 26 had some of their admissions identified, and nine did not have any admission records available [Fig 1].

### Infections: Overall

Both invasive and superficial infections occurred most often in the first 60 days after diagnosis, decreasing in frequency as time from diagnosis increased [Table 3]. Almost two-thirds of patients experienced at least one non-invasive or invasive infection and many patients sustained multiple infections. For the 20 charts that were abstracted in duplicate, there was 100% concordance in the infections identified by the two abstractors.

**Table 3:** Infections by time period after diagnosis.

	Days after acute leukemia diagnosis until death or end of study		
	**0–60 days**	**> 60–100 days**	**> 100–180 days**
**Total patients at risk**	68	48	43
**Total patient days at risk**	3344	1791	2971
**Invasive infections**			
**Total number of patients with** ≥ **1 invasive infection**	26	12	10
**Total invasive infections**	**41**	**14**	**12**
Bacteremia/Candidemia, n (%)	7 (17.1)	1 (7.1)	0
Pyelonephritis	2 (4.9)	0	0
Pneumonia	8 (19.5)	3 (21.4)	6 (50.0)
Cellulitis	10 (24.4)	6 (42.9)	1 (8.3)
Gastroenteritis or colitis	13 (31.7)	4 (28.6)	4 (33.3)
Other (Dengue)	0	0	1 (8.3)
Other invasive- not specified	1 (2.4)	0	0
**Total invasive infections per 100 days at risk**	1.2	0.8	0.4
**Non-invasive Infections**			
**Total number of patients with** ≥ **1 non-invasive infection**	6	3	2
**Total non-invasive infections**	**9**	**3**	**2**
Upper respiratory infection	3 (33.3)	3 (100.0)	0
Otitis media	1 (11.1)	0	0
Infectious stomatitis	2 (22.2)	0	0
*Herpes simplex*	1 (11.1)	0	0
*Herpes zoster*	1 (11.1)	0	0
Candidal dermatitis	1 (11.1)	0	0
Herpes labialis	0	0	1 (50.0)
Ocular	0	0	1 (50.0)
**Total non-invasive infections per 100 days at risk**	0.3	0.2	0.1

^c^There was one infection missing a type in the first 60 days after diagnosis, and this was not included in the calculations for either invasive or non-invasive infections.

Invasive infection: systemic infection or infection of a specific organ system(s)

Non-invasive infections: infections limited to involvement of mucosal barriers

### Invasive infections

**Fig 2:**
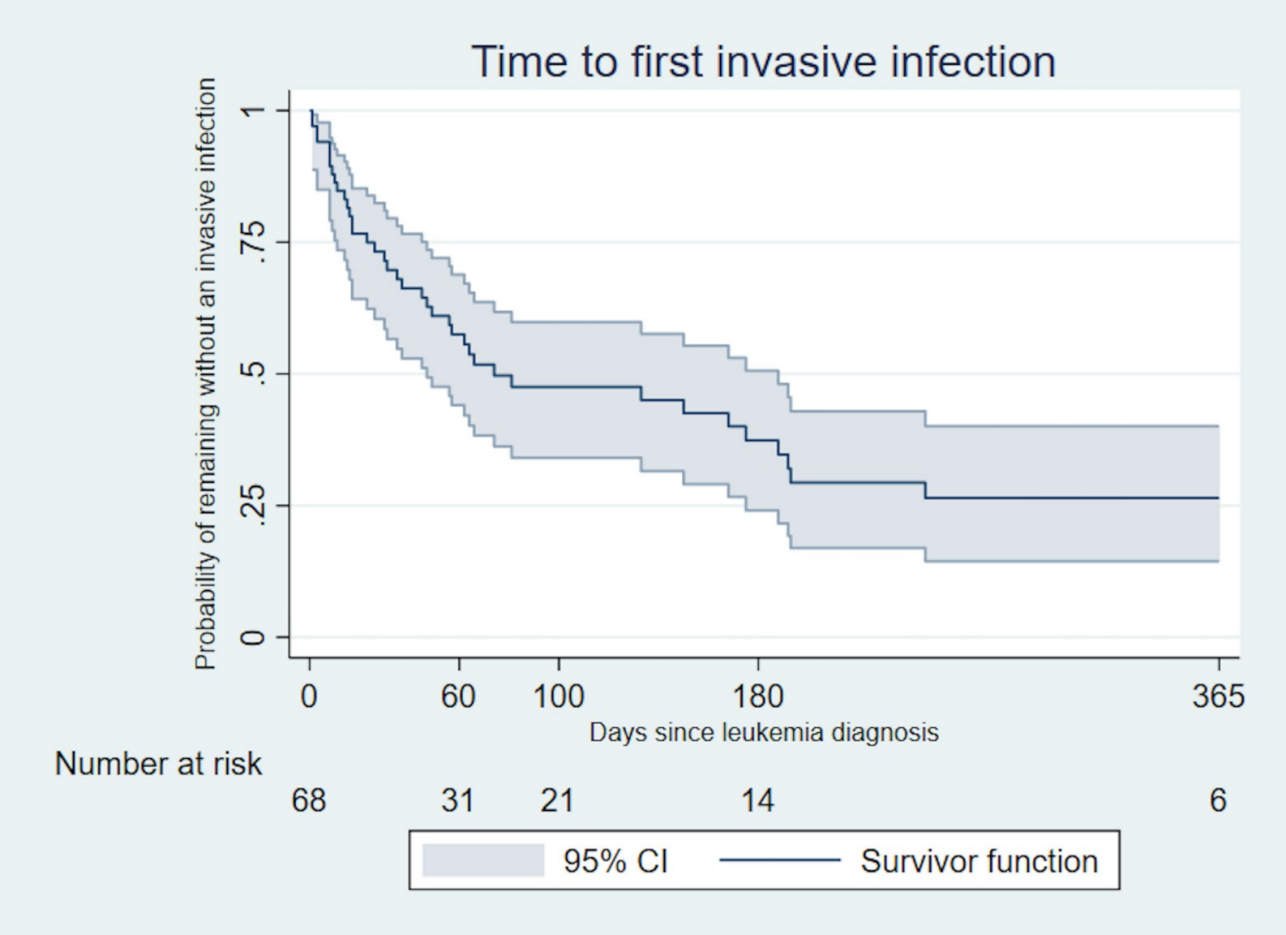
Time to first invasive infection in the first year after diagnosis. Patients were censored at one year, end of study, or death (whichever came first).

There were 1.2 invasive infections per 100 days at risk in the first 60 days after diagnosis, 0.8 from > 60–100 days, and 0.4 from > 100–180 days [Table 3]. A time to first invasive infection curve in the first year after diagnosis highlights the increased risk of invasive infection during the first 60 days [Fig 2]. In the first 60 days when infection rate was the highest, gastroenteritis/colitis, cellulitis, pneumonia, and bacteremia/candidemia were most frequent. Bacteremia/candidemia was less common in the latter periods; however, episodes of pneumonia and gastroenteritis/colitis continued in these later time periods.

The median ANC up to two days before to one day after the date of infection onset was available for 21 infections in the ≤ 60 day period and eight infections in the > 60–100 day period. The median ANC was 636 cells/μL (range 3–4800) and 922 cells/μL (range 10–1419) for these time periods, respectively. The ANC was available for one infection at > 100–180 days and it was 1212 cells/μL.

There were 82 identified inpatient invasive infections during the 2-year study time period (range 0–71, median 0, mean 5.1 days after hospitalization). This did not include 9 infections identified from cause of death review in POND, as admission records surrounding the death were not available. Of these 82 invasive infections, 32 (39.0%) were labeled as hospital-acquired because they started more than two days after admission. Of these 82 infections, 16 (19.5%) occurred more than 7 days after hospital admission.

### Non-invasive infections

There were 0.3 non-invasive infections per 100 days at risk in the first 60 days after diagnosis, 0.2 from > 60–100 days, and 0.1 from > 100–180 days. Non-invasive infections in the first 60 days included upper respiratory tract infections (URI), otitis media, infectious stomatitis, *Herpes simplex* and *Herpes zoster* lesions, and candidal dermatitis. Only otitis media and herpes labialis were identified in the periods of > 60–100 days and > 100–180 days, respectively [Table 3].

### Microbiology diagnostic testing

There were 171 infectious diagnostic tests obtained throughout the study period, of which 96 (56.1%) were cultures for bacteria or fungi, 39 (22.8%) were tests for viruses, and 33 (19.3%) were tests for parasites. There were no cultures obtained specifically for mycobacteria [Table 4].

**Table 4:** Infectious diagnostic testing performed.

	n (%)
Culture for bacteria or fungi	96 (56.1)
Test for viruses	39 (22.8)
Test for parasites	33 (19.3)
Missing source	1 (0.6)
Other^d^	2 (1.2)
**Total**	**171**

^d^PPD (negative), Leptospirosis antibody testing (negative)

The majority of cultures, 80 (83.4%), were blood cultures, and 58 (72.5%) of these were positive. Coagulase-negative *Staphylococcus* was the most common organism. Most of these were not categorized as invasive infections, given lack of central venous catheters, unless characterized in the medical record that they were treated as such. The remaining positive cultures included Gram-negative, Gram-positive, and yeast pathogens. The yield of non-blood culture specimens was limited, except abscess cultures where 4 of 5 cultures had an identified pathogen [Table 5]. Susceptibility results were available for 12 (70.6%) of 17 pathogens identified from blood, urine or abscess cultures [Table 6]. All Gram-negative pathogens with susceptibility testing available were reported as having an extended spectrum beta-lactamase (ESBL) phenotype, and the *Acinetobacter baumannii* isolate was also resistant to carbapenems (imipenem and meropenem), indicating resistance to all beta-lactams. All the *Staphylococcus aureus* isolates were methicillin-resistant *Staphylococcus aureus* (MRSA).

**Table 5:** Source of cultures and results.

	Blood	Urine	Cerebral Spinal Fluid	Abscess Fluid	Other (ear)	Missing Source	Total
**Source of culture**	80	7	2	5	1	1	96
Negative	58	6	2	1	0	0	67
Positive	22	1	0	4	1	1	29
**Organism**							
Coagulase-negative *Staphylococcus*	10				1	1	12
*Staphylococcus aureus*	2			2			4
*Streptococcus* NOS	1						1
*Enterococcus faecalis*	1			1			2
*Acinetobacter baumannii*	1						1
*Enterobacter aerogenes*		1					1
*Klebsiella pneumoniae*	2			1			3
*Pseudomomas aeruginosa*	2						2
*Haemophilus influenzae*	1						1
Gram-negative pathogen not otherwise specified	1						1
*Candida* NOS	1						1

NOS: species not otherwise specified

**Table 6:** Organism resistance profile.

	Resistance testing: Resistance/Intermediate Resistance Profile
*Acinetobacter baumannii*	Blood
	1- **Carbapenem resistant** (resistant to all beta-lactams); also resistant to amikacin, gentamicin, ciprofloxacin
*Enterobacter aerogenes*	Urine
	1- **ESBL**; also resistant to gentamicin, TMP/SMX, norfloxacin, nalidixic acid
*Klebsiella pneumoniae*	Blood
	1- **ESBL;** also resistant to amikacin, ciprofloxacin, gentamicin, TMP/SMX
	2- **ESBL;** also resistant to ciprofloxacin, gentamicin Abscess fluid
	1- **ESBL**; also resistant to ciprofloxacin, gentamicin
*Pseudomomas aeruginosa*	Blood
	1- None reported
	2- None reported
*Haemophilus influenzae*	Blood
	1- None reported
Gram-negative pathogen not otherwise specified	Blood
	1- **ESBL**
*Staphylococcus aureus*	Blood
	1- MRSA (oxacillin resistant);
	2- MRSA (oxacillin resistant); also resistant to erythromycin Abscess fluid
	1- MRSA (oxacillin resistant); no additional sensitivity data
	2- MRSA (oxacillin resistant); also resistant to tetracycline
*Streptococcus* NOS	Blood
	1- None reported
*Enterococcus faecalis*	Blood
	1- Resistant to erythromycin Abscess fluid
	1- Resistant to erythromycin
*Candida* NOS	Blood
	1- None reported

ESBL: extended-spectrum beta-lactamase phenotype (indicates resistant to penicillins, aminopenicillins, cephalosporins, and aztreonam)

NOS: species not otherwise specified

The majority of viral tests obtained were antibody tests from the blood. One dengue virus IgM serology and one herpes simplex virus I/II IgM serology were positive, supporting diagnosis of a current or recent infection [Table 7]. The majority of parasite tests were stool testing for ova and parasites. Of these, 21 (67.7%) were reported as *Entamoeba histolytica* and one for *Blastocystis* species not specified. One ear secretion lead to a diagnosis of myiasis, an infection of fly larvae.

**Table 7:** Viral tests^e^.

Virus tested	Number of tests	Positive
Hepatitis A	3	none
Hepatitis B	4	none
Hepatitis C	5	none
Human immunodeficiency virus	4	none
Epstein-Barr virus (EBV)	5	1 (past infection/immunity)
Cytomegalovirus (CMV)	9	3 (past infection/immunity)
Dengue virus (DENV)	3	1 (current/recent infection: IgM)
Herpes simplex virus (HSV) I/II	4	2 (past infection/immunity HSV I)
		1 (past infection/immunity HSV I, current/recent infection HSV I/II: IgM)
**Total**	**37**	**8**

^e^All blood tests. All antibody tests, except for one nucleic acid test (CMV) that was negative (included in table). There were two tests (EBV and DENV) of unknown test type, both negative (included in table).

### Infection risk factors

**Fig 3:**
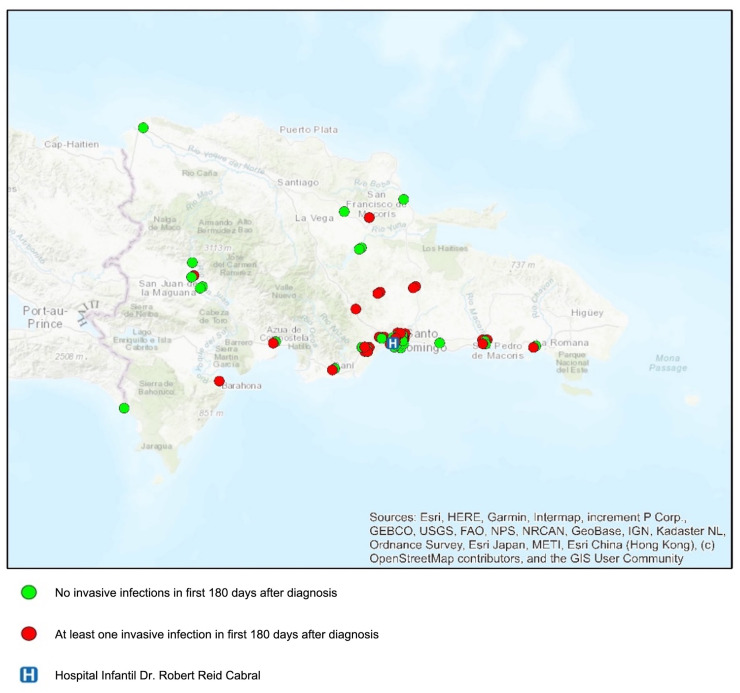
Location of patient residence by city^g^. World topographic baselayer reprinted from ArcGIS Online under a CC BY license. Content is the intellectual property of Esri and is used herein with permission. Original copyright © 2020 Esri and its licensors. All rights reserved [21]. ^g^For three patients where only province was available, location of residence was placed near the center of the province.

In univariate negative binomial regression, age ≥ 10 years (IRR 0.50, 95% CI 0.25–0.99) and distance from the hospital > 100 km (IRR 0.35, 95% CI 0.14–0.90) were each protective against invasive infection in the first 180 days after diagnosis. Both of these risk factors retained significance in a multivariate model [Table 8]. This suggestion of greater distance from the hospital being protective from infection was consistent visually on the ArcGIS map of location of patient residence, which differentiated patients with and without an invasive infection in the first 180 days [Fig 3].

**Table 8:** Risk factors for number of invasive infection in the first 180 days after diagnosis.

	Univariate			Multivariate^f^		
	IRR	95% CI	p-value	IRR	95% CI	p-value
**Acute leukemia type**						
Acute lymphoblastic leukemia	1 (ref)					
Acute myeloid leukemia	1.17	0.47–2.92	0.73			
**Sex**						
Male	1 (ref)					
Female	1.36	0.70–2.64	0.36			
**Age (years)**						
≥ 2 to < 10	1 (ref)			1 (ref)		
≥ 1 to < 2	0.98	0.34–2.81	0.98	0.97	0.36–2.63	0.95
≥ 10	0.50	0.25–0.99	0.046	0.50	0.27–0.91	0.03
**Distance from hospital (km)**						
> 4 to ≤ 30	1 (ref)			1 (ref)		
> 30 to ≤ 100	1.40	0.72–2.71	0.32	1.40	0.74–2.63	0.30
> 100	0.35	0.14–0.90	0.03	0.34	0.14–0.85	0.02
**Purchasing power index**						
≥ 80 to ≤ 120	1 (ref)					
< 80	0.98	0.41–2.30	0.95			
> 120	1.09	0.50–2.37	0.84			
**Weight-for-age percentile**						
≥ 5 to < 85	1 (ref)					
< 5	1.44	0.52–3.99	0.48			
≥ 85	0.91	0.31–2.67	0.86			
Missing	2.65	0.73–9.61	0.14			

^f^Only variables that were significant in univariate analyses were retained in the multivariate model.

IRR, incidence rate ratio; CI, confidence interval; km, kilometer.

Additional univariate negative binomial regression analyses were done focusing on invasive infections in the first 60 days after diagnosis. Again, the point estimates for both age ≥ 10 years and distance from the hospital > 100 km suggested a protective effect of these factors (IRR 0.57, 95% CI 0.23–1.39 for age ≥ 10 years, IRR 0.40, 95% CI 0.12–1.35 for distance > 100 km).

### Clinical outcomes

**Fig 4:**
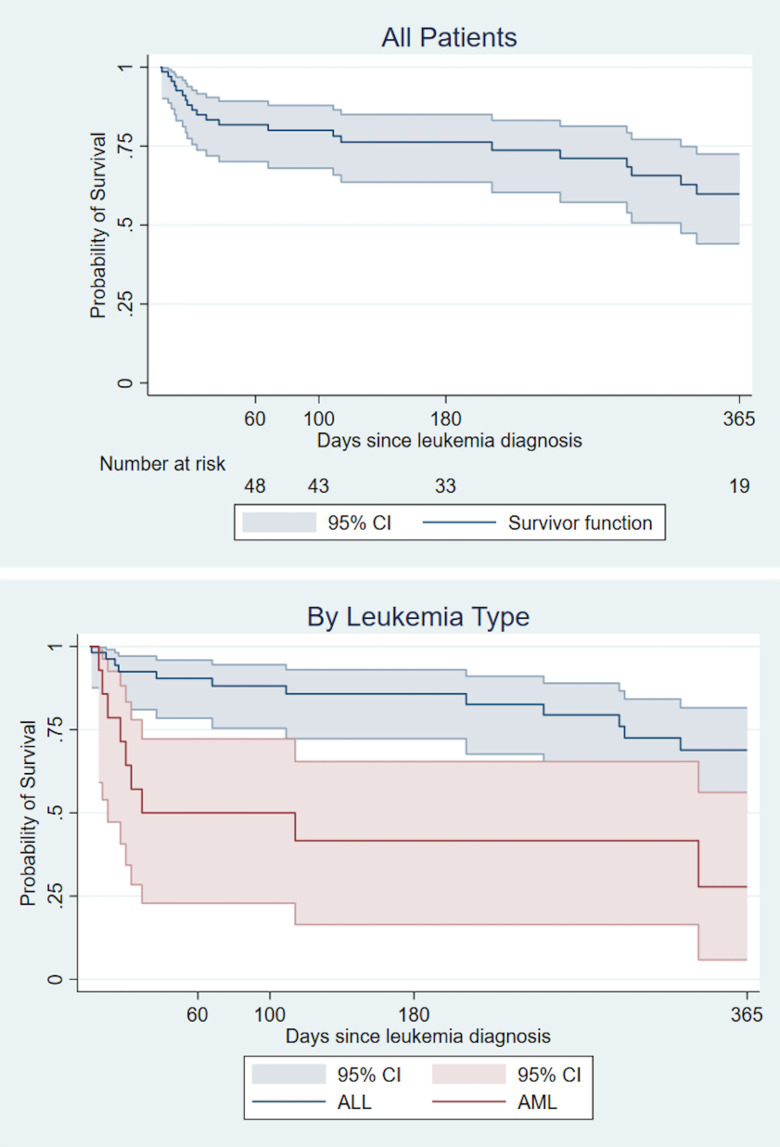
Kaplan-Meier survival estimates in the first year after diagnosis. ALL, Acute lymphoblastic leukemia; AML, Acute myeloid leukemia.

During the two-year study period, 22 patients died at a median of 33 days (range 1–460) after acute leukemia diagnosis. Based on review of medical records for the admission in which the patient died and/or causes of death reported in the POND database, it was determined that eight of the 22 (36%) deaths were related to infection. Survival probability was 81.8% (95% CI 70.1–89.2), 80% (95% CI 68.0–87.9%), and 76.2% (95% CI 63.6–85.0%) at 60 days, 100 days, and 180 days of follow-up, respectively. At the conclusion of one-year follow-up from diagnosis the overall survival was 59.8% (95% CI 44.1–72.5%). The overall survival curve for children with AML was significantly worse compared to the survival curve for those with ALL (log-rank test for equality of survivor functions: Χ^2^ 11.69, p-value 0.001) [Fig 4].

## Discussion

Invasive infections were common and a prominent source of death in this cohort of patients with newly diagnosed ALL and AML treated at HIRRC in Santo Domingo. Rates of invasive infections were highest in the first 60 days after diagnosis, a time period that may be a target for interventions. Furthermore, although data are not complete, antibiotic resistant pathogens were essentially universal among the bacteria cultured from blood, urine and abscesses. The baseline factors of older age (≥ 10 years) and farther distance from the hospital (> 100 km) were each negatively associated with invasive infection in the first 180 days after acute leukemia diagnosis.

Previous studies have highlighted the difference in survival rates from pediatric acute leukemia between HIC versus LMIC [3, 11]. Children and adolescents with ALL enrolled in Children’s Oncology Group clinical trials from 2000–2005 in the U.S. and Canada had five-year survival rates reaching 90% [12], whereas those treated on a cooperative study protocol across several Central American countries from 2008–2012 had a three-year survival rate of 68% [13]. Among the current cohort, we found a similarly reduced survival probability for patients with ALL at one year. Overall survival was worse in the AML subset of patients as would be expected given generally poor prognosis of AML compared to ALL.

Not only was the mortality rate in this cohort high but the time to death after acute leukemia diagnosis was also particularly rapid at a median of 33 days. Approximately one-third of these deaths were attributed to infection. This is likely an underestimate of attribution of mortality to infection as medical record data of the death was unavailable for most patients, and information in POND on cause of death was limited. Nonetheless, the findings that deaths were early and often infection-related are consistent with that described in other pediatric acute leukemia cohorts in Latin America and LMIC settings worldwide. In a study of children (0–16 years) in El Salvador with a diagnosis of ALL or AML between 2000–2007, infection was the most common cause (60–70%) of treatment-related mortality [14]. High rates of infection-related mortality have also been described in other ALL and AML cohorts from El Salvador, Guatemala, and Honduras, with the majority of deaths occurring before or during induction [15, 16]. A more recent study inclusive of patients from Costa Rica, El Salvador, Honduras, Nicaragua, and Panama enrolled in a cooperative group trial (AHOPCA-ALL 2008) from 2008 to 2012 reported that infection accounted for 64% of all deaths in induction or during remission [13]. In these studies, specific types of infections and overall infection rates for patients in the cohorts were not specified. Therefore, our study makes a unique contribution towards descriptive epidemiology of infectious complications in pediatric acute leukemia patients in Latin America.

The number of microbiological cultures obtained in our cohort was relatively low given the number of hospitalizations. The low number of cultures obtained likely contributed to a limited number of pathogens identified as the source of an infectious event. Despite the limited pathogen detection, the resistance profile for the identified Gram-negative and Gram-positive pathogens was striking. In particular, all isolates of *S*. *aureus* were MRSA and all Gram-negative pathogens with susceptibility testing had an ESBL phenotype and/or were resistant to a carbapenem. Additionally, a number of these organisms met criteria for multi-drug resistance as defined by the Centers for Disease Control and Prevention [17].

In 2016, the HIRRC hospital-wide antibiogram revealed 81% resistance to 3^rd^ and 4^th^ generation cephalosporins across 49 *Klebsiella pneumoniae* blood isolates, as well as 60% resistance to 3^rd^ generation and 50% resistance to 4^th^ generation cephalosporins among five *Enterobacter aerogenes* blood isolates. Furthermore, among the 95 *S*. *aureus* blood isolates, 70.2% resistance to oxacillin was reported. Therefore, the resistance profiles of the pathogens identified in our acute leukemia cohort appear to reflect the high resistance rates present in the general HIRCC patient population. These findings suggest a need for antimicrobial stewardship both at the hospital level and within the oncology population to reduce further evolution of resistance, as well as continued optimization of empiric antibiotic therapy for suspected infection.

The types of infection and level of immunosuppression were also of interest. While pneumonia and bloodstream infections were relatively common in the first 60 days post-diagnosis, gastroenteritis/colitis and cellulitis were more common. Gastroenteritis/colitis cases were often associated with a report of *Entamoeba histolytica* on stool ova and parasite testing. The increased frequency of cellulitis was hypothesized to be related to lack of standardized procedures for peripheral IV (PIV) placement. Both of these sources of infection need to be confirmed by additional observation, but each could be targeted by implementation of standardized procedures. Adapted treatment regimens that decrease chemotherapy intensity or modify staging and risk stratification have been used in LMIC to maximize cure rates and minimize toxicity [9, 18, 19]. As these protocols are refined, supportive care measures will also need to be adapted to reduce infection risk. For example, stool screening for parasites and treatment if indicated could be implemented in advance of chemotherapy to reduce downstream events of gastroenteritis/colitis. Notably, the relatively high ANC in our cohort at the time of infection further argues for improving supportive care, given the number of infections that developed in the absence of severe neutropenia (ANC < 500).

Although a clear majority of invasive infections started in the community, a substantial proportion of infections occurred more than two days after hospitalization. While applying this blanket dichotomy to classify community versus hospital-acquired infections may not capture the nuances within this heterogeneous group, it broadly highlights the importance of supportive care practices that target both inpatient and outpatient settings.

The association of older age (≥ 10 years) and farther distance from the hospital (> 100 km) with a decreased incidence of invasive infection in the first 180 days was unexpected. Regarding older age, although we initially assumed older children may have higher risk acute leukemia and thus be at greater risk of severe infection, it may be that they have had more time to develop immunity to common infections, have better hygiene, and/or are more nutritionally replete, resulting in an overall reduced infection risk.

Anticipated longer travel time to the hospital and low socioeconomic status (annual household income < $2000 USD) were both found to be independent risk factors for infection-related deaths in a prospective cohort study of children newly diagnosed with ALL and AML in El Salvador [20]. Therefore, we hypothesized that patients whose home residence was farther from the hospital would have a greater risk of invasive infection requiring hospitalization, as the increased distance would likely equate to increased travel time required to access pediatric oncologic specialty care. In terms of farther distance from the hospital being a protective factor from severe infection in our study, we propose two possible explanations. First, this may be related to incomplete capture of infectious events. If patients that lived a further distance from the hospital developed an invasive infection, they may not have been able to return to HIRRC for care. Second and more likely, is that this association was mediated by the presence of a guest-house for patients close to the hospital, Casa FACCI (La Fundación Amigos contra el Cáncer Infantil). Casa FACCI provides exemplary clean accommodations along with a consistent opportunity for adequate nutrition. This house is likely to have been preferentially utilized by families living further from the hospital and may have helped reduce the risk of exposure to pathogens and/or allow prompt medical attention at the onset of illness. One strategic intervention to reduce infection rates may be to identify patients at the greatest risk, especially those < 10 years, and house them at Casa FACCI during periods of anticipated neutropenia within the first 180 days after diagnosis, pending space and resource availability.

### Limitations

Our findings must be interpreted within the context of a number of limitations. First, although our study captured all newly diagnosed acute leukemia patients within a two-year period at HIRRC, the resultant cohort was still relatively small. This reduced the stability of point estimates for risk factors assessed and reduced the power to identify statistically significant risk factors for infection.

Second, the reliance on retrospective chart abstraction likely resulted in an underestimation of the number of infections in this cohort, as well as infection-related deaths. In reality, both may be much higher. Charts were unavailable for almost 16% of the total admissions identified, and the majority of inpatient deaths had limited documentation. Patients may have sought care at other local hospitals for infection which could have further led to underestimation of infection rates; however, this was thought to be rare given that HIRRC is one of the primary pediatric hospitals in the country and one of the few with oncology expertise. All microbiology data capture was reliant on information in the medical record or Oncology culture logbook. While record books of all hospital cultures kept in the microbiology laboratory allowed for searching of missing information about a specific culture (e.g. susceptibility data), the ability to identify all cultures obtained in the Oncology unit from the microbiology laboratory logbooks was limited. Finally, there were limited data on family-level resources and so association of reduced family resources with infection risk could not be fully explored.

## Conclusions

Despite the limitations, this study highlights the possibility to perform a retrospective cohort study in a LMIC to establish data for a granular description of the types of infection, timing of onset, and outcomes. As this cohort was inclusive of all patients at the largest referral center for pediatric acute leukemia in the Dominican Republic, the data can be informative to implementing quality improvement initiatives to reduce infection. Future interventions should focus on children < 10 years and should leverage the community housing resource, Casa FACCI, close to the hospital to prevent community-acquired infections. Additionally, the high rates of gastroenteritis/colitis and cellulitis should prompt attempts for implementing practices to screen for parasites at diagnosis and to standardize PIV placement. Finally, the suggestion of a high proportion of resistant organisms and number of hospital-acquired infections warrants consideration of further antimicrobial stewardship and infection prevention and control initiatives.

This work has served as an important first step in developing a collaborative research effort between CHOP and HIRRC and establishing a foundation to guide future work. Strategies that are effective in reducing infection-related mortality at HIRRC can be shared with other pediatric oncology providers in the Dominican Republic and Latin America.
